# Spatial and Socioeconomic Inequalities in Accessibility to Healthcare Services in South Korea

**DOI:** 10.3390/healthcare10102049

**Published:** 2022-10-17

**Authors:** Sangwan Lee

**Affiliations:** Department of Urban Planning and Engineering, Hanyang University, 206, Wangsimni-ro, Seongdong-gu, Seoul 04763, Korea; esangwan@hanyang.ac.kr

**Keywords:** transport disadvantage, opportunity inequality, accessibility, healthcare services, nationwide spatial analysis

## Abstract

This study explored questions of (1) whether certain areas of South Korea experienced inequal accessibility to public health centers, private hospitals/clinics, and general hospitals by car and public transportation using gaussian mixture models (GMM) and (2) whether socially disadvantaged socioeconomic groups faced disproportionate burdens on accessibility to the multi-tier healthcare services employing ordinary least square regression models (OLS). This study used nationwide accessibility indicators in South Korea measured by Korea Transport Institute in 2019. The main findings were as follows: First, the results of the GMM indicate that the degree of accessibility to healthcare services was significantly lower in rural, mountainous, and seaside locations compared to metropolitan areas. Second, there was more considerable inequality in public transportation accessibility than car accessibility. Third, the findings of the OLS reveal a significant relationship between accessibility indicators and socioeconomic variables, such as age, gender, disability, and residential location, which indicates socioeconomic inequality in accessibility in South Korea. This study contributes to shedding light on understanding the spatial and socioeconomic inequality in accessibility across the nation and offering policy implications.

## 1. Introduction

Accessibility to healthcare services is a critical component [1] since easier access to the services leads to a variety of positive outcomes, such as more regular visits and improvements in overall health [2,3]. Theoretically, the egalitarianism framed by Rawls [4] argues that transportation projects ought to be motivated by respect for the basic rights of persons [5]. The application of Rawls’ difference principle to the transportation field implies that for plans and policies to be considered fair, they should improve the levels of accessibility experienced by marginalized population groups, ensuring that at least the basic needs essential to a decent life are met [6]. Unfortunately, this is not the case in practice due to various factors, such as the inequal allocation of healthcare facilities and staffs [7]. Most of us confront some type of inequal accessibility to healthcare services in our daily lives; even, the degree to which this occurs may vary across individuals in different spatial and socioeconomic contexts [8].

The empirical research that lies at the intersection of accessibility and equity has been thriving [9,10] since seminal works by Wachs and Kumagai [11] and Banister [12]. Previous literature has identified some degree of inequal accessibility to healthcare services, and it varies across individuals in different spatial and socioeconomic contexts [8,13]. For instance, Steinberg et al. [14] found that people with disabilities had difficulty accessing healthcare services in three U.S. cities. Dai [15] indicated that a high percentage of people in disadvantaged populations, such as those with a low socioeconomic level or who face sociocultural hurdles, are likely to have high rates of late diagnosis in Detroit, Michigan. Bissonnette et al. [16] revealed lower potential accessibility for linguistic minorities and recent immigrant communities to healthcare services in Mississauga, Ontario, Canada. A study by Granbom et al. [17] demonstrated older adults in Sweden faced substantial accessibility issues and a high prevalence of environmental barriers. In addition, a body of research has investigated the issue in developing countries [18,19] since the issue in the nations is of significance and usually severe [9]. For instance, Mokomane et al. [20] explored South Africa and concluded that structural and systemic problems continued to impede equitable accessibility. In addition, Kuupiel et al. [21] discovered inequal accessibility to public health facilities that offer TB testing services in Ghana. Few prior research has engaged on this topic in the South Korean context; for instance, Yoon and Park [22] measured accessibility by public transportation to primary care services for older adults using a case study of Seoul and revealed spatial inequality.

This study identified and filled the following research gaps: (1) few existing studies have conducted a nationwide analysis, (2) few have comprehensively explored spatial and socioeconomic-based inequality in accessibility to different types of healthcare services, (3) few have studied inequal accessibility to healthcare services in the South Korea context, and (4) few have focused on accessibility by both private car and public transportation. Therefore, the research aims to fill the gaps and answer two lines of questions. Thus, this study aims to answer two research questions. The first line of question is whether there were spatial variations of accessibility to healthcare services by car and public transportation across South Korea. The second line of questions concerns whether marginalized population groups in South Korea (e.g., women, individuals with disabilities, elderly adults, beneficiaries, and immigrants) significantly took longer to access multi-tier healthcare services. This study employed gaussian mixture models (GMM) to answer the first research question and ordinary least square regression models (OLS) to explore the remaining question using the nationwide accessibility matrix measured by Korea Transport Institute in 2019. This research has three objectives: (1) shedding light on the knowledge of spatial and socioeconomic disparities in accessibility across the country, (2) identifying certain areas and socioeconomic groups that require accessibility improvements, and (3) drawing policymakers’ attention and providing policy implications.

## 2. Materials and Methods

The procedure of this study is as follows. First, this study chose South Korea as a case study area and gathered relevant data. Second, this study employed GMM to address the first research question by classifying and visualizing healthcare service accessibility by car and public transportation throughout South Korea. Third, this study developed OLS to examine the associations between accessibility and socioeconomic features. Each stage is described in depth in the following subsections.

### 2.1. Study Area

The study area of the paper was the whole country of South Korea, with a population of approximately 51 million persons in an area of around 100,000 km^2^. Eight provinces, seven metropolitan regions (e.g., Seoul and Busan), and one special self-governing city (i.e., Sejong) make up the major administrative divisions in South Korea. Administratively, South Korea can be divided at the further finer levels, including Eup/Myeon/Dong (EMDs). According to the Healthcare Bigdata Hub, there were around 94,865 hospitals in South Korea in 2019. This number included approximately 350 general hospitals, approximately 52,000 private hospitals, and clinics, and approximately 3500 public health facilities.

### 2.2. Data

This study used the accessibility indicator across the country measured by Korea Transport Institute in 2019. The accessibility indicator used in this study was the average travel time from the centroid of a EMD by weight based on the total floor area of buildings to healthcare services within the EMD boundary. It is important to note that the accessibility measure employed a relatively straightforward approach, although a body of previous literature has developed quantitative measures of accessibility [23]. For instance, Hansen [24] proposed gravity measures, Luo and Wang [25] employed the two-step floating catchment area (2SFCA) method, and Tao and Wang [13] applied the Gaussian 2SFCA. Travel time to the closest destination, however, has proven to be an adequate indicator since it is not challenging to put into practice and offers a direct interpretation in absolute units, which, in turn, makes it possible for decision-makers to be easily understood [9]. Furthermore, the accessibility was not to detailed aspects of healthcare services, such as specialists or emergency departments, but to three types of facilities, including general hospitals, private hospitals and clinics, and public health centers.

This study benefits from using the data set for the following reasons. First, the accessibility was significantly improved by applying additional nuances according to the type of healthcare service, the time of travel, and the transportation modes. Specifically, it considered various types of healthcare services, which were (1) general hospitals, (2) private hospitals and clinics, and (3) public health centers. The laws and regulations were used to classify the various types of healthcare services based on diverse factors, such as the purpose of the services, size, and facilities, an example of each category is presented in Table 1. The accessibility considered three separate times of travel: (1) morning peak (7 am to 9 am), (2) daytime (12 pm to 14 pm), and (3) evening peak (18 pm to 20 pm). The final nuance involved using a car and public transportation to evaluate the accessibility of different modes of transportation. Second, the data set measures nationwide accessibility to healthcare services. Much of previous research has explored certain metropolitan areas or cities, which may produce a selection bias [26]. However, this study used the data set to avoid selection bias. Third, given COVID-19 has disrupted transportation aspects in South Korea [27,28], accessibility measures in 2019, which was before the pandemic outbreak, may not cause validity issues in the findings. Fourth, accessibility was assessed at the finer administrative unit of EMDs.

### 2.3. Analytic Methods

This study employed two methods to answer the two research questions adequately: (1) GMM and (2) OLS. Subsections present details on the analytic methods.

#### 2.3.1. Gaussian Mixture Model

This study used GMM for the following reasons. First, it helps find hidden patterns in the dataset and then classify instances into a certain number of clusters [29,30]. In this case, GMM allows to detect clusters with different accessibility indicators listed in Table 2 and classifies EMDs into a certain cluster [31]. Second, the limitation of the k-means clustering algorithm, such as non-probabilistic aspects and lack of flexibility in cluster shapes, leads to poor performance in many real-world situations [32]. Thus, this study used a sophisticated clustering algorithm, GMM. Therefore, GMM has been the subject of a significant amount of research due to its effectiveness and efficiency [33].

This study developed two GMM algorithms to explore spatial clusters that differed in accessibility by two modes of transportation (i.e., car and public transportation) since accessibility by car and public transportation generally differ from one another [34]. Thus, this study generated one set of clusters with a focus on accessibility by automobile using Access 1 to 9, and another set of clusters with a focus on accessibility by public transportation using Access 10 to 18 (see Table 2).

#### 2.3.2. Ordinary Least Square Regression Model

This study developed 18 OLS models. This study used each accessibility indicator listed in Table 2 as a dependent variable in each OLS model. This paper did not use clusters identified in GMM as a dependent variable and develop discrete choice modeling since it attempted to explore the distinction in estimates of the independent variables between the different times of day, such as the morning peak, and types of healthcare services, such as general hospitals.

The final model specifications include seven independent variables (see Table 3) gathered from different population registers provided by Statistics Korea. The set of independent variables was selected based on previous literature. For instance, Dodson et al. [35] conducted an extensive literature review and operationalized transport disadvantaged, which are low-income people, the unemployed, beneficiaries, women, the elderly, disabled, and ethnic minorities. Furthermore, Blumenberg [36] found the lack of accessibility women and low-income workers faced. Moreover, Lucas [37] indicated where a person lives could prevent them from accessing transport services, such as in rural areas or on peripheral urban estates. Lee et al. [38] revealed immigrants in the U.S. showed different travel behavior compared to U.S.-born residents.

The dependent and independent variables used the log transformation, which overcomes issues due to their skewed distributions and offers estimates of the constant elasticity effect of independent variables on the dependent variable [39].

## 3. Results

This section first presents the spatial inequality in healthcare accessibility by car and public transportation in South Korea using GMM, and then it demonstrates socioeconomic inequality in accessibility to healthcare services employing OLS.

### 3.1. Spatial Inequalities in Accessibility to Healthcare Services

**Figure 1:**
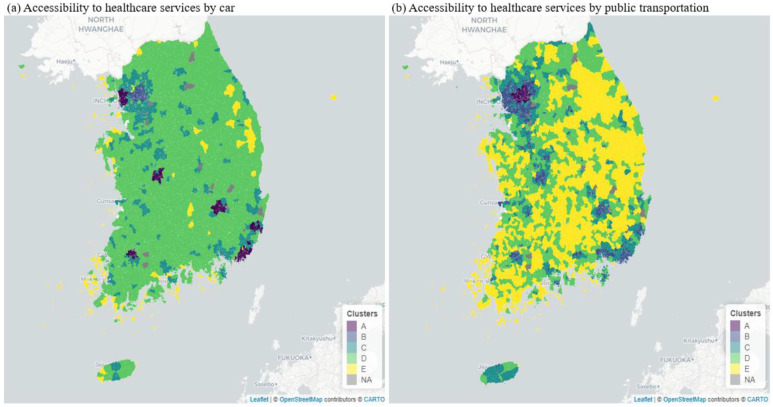
Using the two Gaussian Mixture Models to Find Five Clusters (Cluster A: highly accessible areas; Cluster B: accessible areas; Cluster C: moderate accessible areas; Cluster D: inaccessible areas; Cluster E: highly inaccessible areas). (**a**) Accessibility to healthcare services by car; (**b**) Accessibility to healthcare services by public transportation.

Figure 1 visualizes the spatial distribution of accessibility to multi-tier healthcare services, including general hospitals, private hospitals/clinics, and public health centers in South Korea based on GMM. EMDs, which are finer administrative units in South Korea, were clustered into clusters based on the accessibility measures listed in Table 2. This study developed one set of clusters to investigate car accessibility (see Figure 1a), and another set of clusters to explore public transportation accessibility (see Figure 1b). When selecting the number of clusters (i.e., components), this study used the Akaike information criterion (AIC) or the Bayesian information criterion (BIC). The results of the AIC and BIC indicated that the optimal number of clusters was 5 in both car and public transportation accessibility. Thus, the fitted GMM has a number of clusters of 5 with the full covariance, which allows each cluster to be modeled as an ellipse with arbitrary orientation. Additionally, Table 4 and Table 5 present the average travel time to healthcare services by car and public transportation in each of the five clusters.

Two major findings are as follows. First, the results in Figure 1 demonstrate spatial inequality in accessibility to multi-tier healthcare services in South Korea. Table 4 and Table 5 also indicate how significant spatial variation in the accessibility between the groups is, given the *p*-values of the ANOVA tests. Specifically, Cluster A showed the highest accessibility to healthcare services, which was concentrated in Metropolitan areas, including Seoul, Incheon, Daejeon, Daegu, and Busan. Higher healthcare accessibility by car and public transportation was also clustered in Cluster B immediately surrounding Cluster A. However, accessibility levels considerably decreased in the rural, mountainous, and coastal areas, identified as Cluster D and E. Specifically, residents in Cluster E generally encountered considerable burdens in terms of travel time to the three types of healthcare services. For instance, they take around 80 to 90 min to access general hospitals by car, while those in Cluster A take less than 10 min.

Second, Figure 1a,b illustrate that the spatial inequality in accessibility significantly differed between cars and public transportation. Specifically, the findings shown in Figure 1b suggest that when considering the accessibility of public transportation, the spatial inequality in accessibility to healthcare became more apparent, particularly in the rural, mountainous, and coastal areas. For instance, Table 4 and Table 5 indicate that the average travel time to public health centers, private hospitals/clinics, and general hospitals by car was 10.39-, 53.90-, and 81.66-min during the daytime, respectively. However, when taking public transportation, the average travel time to reach the three different types of healthcare services was 45.03, 84.86, and 111.47 min, respectively. For example, when it comes to obtaining healthcare services, those who live in the cluster that has the poorest accessibility should be required to spend a disproportionally far longer total amount of time traveling especially when using public transportation.

### 3.2. Socioeconomic Inequalities in Accessibility to Healthcare Services

This study conducted further analyses to explore the ways in which the spatial layout aspects of accessibility to healthcare services related to the socioeconomic factors of EMDs. Thus, this study used OLS to capture the association between the degree of accessibility to multi-tier healthcare services, including general hospitals, private hospitals, and public health centers, and the socio-economic factors that represent transportation disadvantages. The results of the 18 OLS models are displayed in Table 6 and Table 7; overall, the fit of the model is good given the R-squared and adjusted R-squared. Table 6 shows socioeconomic inequality in accessibility to healthcare services by car, while Table 7 reveals that by public transportation. This subsection interprets key findings.

First, the findings of Table 6 demonstrate the statistically significant associations between travel time to healthcare services by car and multiple transportation disadvantage factors. Unexpectedly, this study found that not all transport disadvantages did have a significant and positive association with inaccessibility to the three types of healthcare services in South Korea. For instance, age, gender, disability, and residential location were factors that played a significant influence in OLS models 1 to 9. Specifically, OLS 1, 2, and 3 in Table 6 indicate that the elderly were more likely to perceive considerable inaccessibility to general hospitals by car during both morning peak, daytime, and evening peak, while holding other variables constant. Average travel time to the general hospitals during the morning peak increased by 37.2% if the elderly population density at EMDs increased by 1% when everything else was equal. Expectedly, persons in rural areas had considerably limited accessibility to general hospitals and private hospitals, whereas accessibility to public health centers was better in rural areas. If EMDs are in rural areas, the average travel time to private hospitals/clinics will increase by around 110.3% for accessibility to general hospitals and 116.7% for accessibility to private hospitals during the daytime. Furthermore, this study confirms people with disability and females were still part of systemic social inequality in terms of the accessibility to public health centers by car. Interestingly, immigrants had fairly equitable accessibility to health services.

The socioeconomic inequality in accessibility to the three types of healthcare services via public transportation in South Korea is outlined in Table 7. OLS models 10 to 18 in Table 7 reveal that accessibility to healthcare services was hampered by socioeconomic features, such as age and residential location. The estimate variables indicate that the healthcare service accessibility was positively and significantly associated with the density of the elderly controlling for the other variables. EMDs with a higher elderly population density tended to have lower accessibility to private hospitals/clinics through public transportation, given the significant and positive coefficients of 0.092, 0.184, and 0.264 in OLS models 13, 14, and 15. Furthermore, results of OLS 18 show the elderly faced public transportation inaccessibility to public health centers during the evening peak. These findings also indicate that inaccessibility was intensified during the evening peak compared to the morning peak and daytime. Interestingly, despite the insignificant coefficients of the immigrants in models 1, 2, and 3 in Table 6, significant coefficients were found in models 10, 11, and 12 in Table 7. This indicates that the immigrants experienced inequal accessibility by not car but public transportation to the upper-tier healthcare service (i.e., general hospitals) in South Korea. Moreover, OLS 16 and 17 in Table 7 found significant and positive associations between the density of people with disabilities and average travel time to public health centers. Based on Model 16, an increase in the density at EMDs by 1% was associated with an increase in travel time to public health centers by 16.8%, on average. Lastly, living in rural areas also contributed to higher inaccessibility to all three types of healthcare services by public transportation, while, interestingly, the residential location had better accessibility to public health centers by car (see OLS 7, 8, and 9).

## 4. Discussions

### 4.1. Main Findings

With the clustering (GMM) and regression analyses (OLS) taken together, the empirical findings of this study revealed spatial and socioeconomic inequality in accessibility to three types of healthcare services (i.e., general hospitals, private hospitals/clinics, and public health centers) by car and public transportation throughout South Korea. Several key findings were as follows. First, the results of GMM reveal that the accessibility level significantly decreased in the rural, mountainous, and seaside areas, identified as Cluster D and E. Residents in Cluster E generally faced considerable burdens in travel time to the three types of healthcare services; for instance, they take around 90 min to access general hospitals by car, while those in Cluster A take less than 10 min. Second, the spatial variation was considerably different between car and public transportation; more precisely, it is evident that there was a more prevalent disparity in public transportation accessibility in South Korea. Third, the findings of OLS confirmed socioeconomic inequality in accessibility to the three types of healthcare services, although not all transport disadvantages did have a significant and positive association with inaccessibility to the three types of healthcare services in South Korea. For instance, EMDs with a higher elderly population density had significantly lower accessibility to general hospitals and private hospitals/clinics by both car and public transportation. Furthermore, immigrants experienced inequal accessibility by not car but public transportation to the upper-tier healthcare service (i.e., general hospitals) in South Korea. Additionally, people with disability and females were still part of systemic social inequality in terms of the accessibility to public health centers by car. Moreover, persons in rural areas had considerably limited accessibility to general hospitals and private hospitals, whereas accessibility to public health centers was better in rural areas

### 4.2. Policy Implications

This study offers policy implications based on the findings. The first step in addressing the accessibility issue should be to establish some guiding principles. A robust ethical foundation supports this since freedom of movement and equal accessibility access to essential destinations are basic universal human rights [8]. Furthermore, accessibility in the sense of being able to access key destinations should be considered a basic capability due to its central role in enabling people to satisfy basic needs [5,40]. In this context, it is imperative that those with low incomes, women, people with disabilities, older adults, and members of minority groups be included in the planning process and the policy design of urban and transport system policies. In sum, given that decisions regarding transportation planning will inevitably produce costs and benefits that vary across different communities [3], policies should ensure that individuals have at least some level of accessibility to those key activities that are necessary for satisfying their basic needs and daily lives.

Furthermore, accessibility is a major concern in healthcare policy [41] due to its influence on health behavior and outcome [42] since certain population groups may experience a disproportionately detrimental impact as a result of spatial and socioeconomic inequality in accessibility to healthcare services [9]. From the point of view of the allocation of healthcare services, new healthcare facilities, particularly private hospitals/clinics, and general hospitals, should be added to rural areas and areas with a higher proportion of transport disadvantages.

Moreover, several strategies in transportation planning are worth enhancing accessibility. For instance, areas with a higher density of transport disadvantages should have access to a variety of means of transportation, including local access initiatives, community transport, and a paratransit system [43]. Beyond the pre-existing system, private firms, such as Uber, might be encouraged to fill the accessibility gaps that public transit services are unable to fill [34]; for instance, ridesharing services, such as Uber, can be promoted to fill the temporal and spatial gaps that public transit services cannot fill. For instance, the Pinnellas Suncoast Transit Authority in Florida has initiated a program that partners with Uber to provide free trips to low-income neighborhoods [44]. Furthermore, informal transport services, such as minibusses, vans, three-wheelers, and motorcycles, may be able to bridge the gaps in transportation because these modes provide important benefits, including on-demand access to healthcare services and expansion of service coverage in areas that do not have formal transit, particularly to the transport disadvantages [45]. Lastly, another strategy would be to optimize both bus and rail schedules to improve the accessibility of public transportation particularly in rural areas. Because they may not be able to buy a car or one of the other modes of transportation discussed earlier, financial support is the fourth factor that can play a significant role in improving accessibility for the transport disadvantaged.

### 4.3. Limitation and Future Research Direction

This study acknowledges several limitations. First, this study used a relatively simple accessibility measure, although various sophisticated formulations of accessibility have been proposed [46]. Second, the spatial unit (i.e., EMDs) used in this study was somewhat coarse. Third, this study did not examine the complex interplay between intrinsic (i.e., individual attributes) and extrinsic factors (i.e., built environment factors and sociocultural conditions) [8]. Fourth, the findings of a case study conducted in other nations with a different setting may differ from those reported in this study. Fifth, this study did not explore the accessibility to specialists.

Furthermore, this study outlines several potential avenues for further investigation. Given accessibility is a crucial element of the quality of life [11], future research is needed to offer empirical evidence for this connection between the two concepts. Further studies should explore the minimum levels of accessibility to key destinations and the extent to which policies respect individual rights.

## 5. Conclusions

This paper explored spatial and socioeconomic inequality in accessibility to multi-tier healthcare services, including general hospitals, private hospitals/clinics, and public health centers, by car and public transportation in South Korea. Using nationwide data set offered by Korea Transport Institute in 2019, this study employed two analytic methods: (1) GMM to classify and visualize inequal and spatial accessibility to healthcare services for car and public transportation across South Korea and (2) OLS to examine whether certain socioeconomic population groups in South Korea (e.g., women, individuals with disabilities, elderly adults, beneficiaries, and immigrants) experienced inequal accessibility to multi-tier healthcare services. The contributions of this study are three-fold. First, it sheds light on understanding the spatial and socioeconomic inequality in healthcare accessibility throughout South Korea using the nationwide analysis. Second, this study pinpoints geographic areas as well as subsets of the population groups that require additional accessibility enhancements in the healthcare system. Third, the findings of this research will garner significant attention and offer policy implications.

## Figures and Tables

**Table 1:** Examples of the Three Types of Healthcare Services Explored in this Study (Photo by Sangwan Lee).

General Hospital	Private Hospital/Clinic	Public Health Center
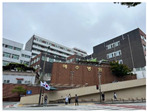	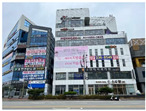	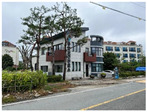

**Table 2:** Description of Variable Used in Gaussian Mixture Models.

Variables	Description	Mean	S.D.
**Cluster Analysis for Accessibility to Healthcare Services by Car**			
Access 1	Log-transformed averaged travel time to general hospitals within the EMD boundary by car during morning peak (7 am–9 am) in minutes	2.57	0.93
Access 2	Log-transformed averaged travel time to general hospitals within the EMD boundary by car between 12 pm and 14 pm in minutes	2.62	0.92
Access 3	Log-transformed averaged travel time to general hospitals within the EMD boundary by car during evening peak (18 pm–20 pm) in minutes	2.64	0.92
Access 4	Log-transformed averaged travel time to private hospitals and clinics within the EMD boundary by car during morning peak (7 am–9 am) in minutes	1.35	0.96
Access 5	Log-transformed averaged travel time to private hospitals and clinics within the EMD boundary by car between 12 pm and 14 pm in minutes	1.39	0.95
Access 6	Log-transformed averaged travel time to private hospitals and clinics within the EMD boundary by car during evening peak (18 pm–20 pm) in minutes	1.41	0.96
Access 7	Log-transformed averaged travel time to public health centers within the EMD boundary by car during morning peak (7 am–9 am) in minutes	1.86	0.43
Access 8	Log-transformed averaged travel time to public health centers within the EMD boundary by car between 12 pm and 14 pm in minutes	1.90	0.43
Access 9	Log-transformed averaged travel time to public health centers within the EMD boundary by car during evening peak (18 pm–20 pm) in minutes	1.92	0.46
**Cluster analysis for accessibility to healthcare services by public transportation**			
Access 10	Log-transformed averaged travel time to general hospital within the EMD boundary by public transportation during morning peak (7 am–9 am) in minutes	3.45	0.93
Access 11	Log-transformed averaged travel time to general hospital within the EMD boundary by public transportation between 12 pm and 14 pm in minutes	3.51	0.96
Access 12	Log-transformed averaged travel time to general hospital within the EMD boundary by public transportation during evening peak (18 pm–20 pm) in minutes	3.55	0.98
Access 13	Log-transformed averaged travel time to private hospitals and clinics within the EMD boundary by public transportation during morning peak (7 am–9 am) in minutes	2.44	1.26
Access 14	Log-transformed averaged travel time to private hospitals and clinics within the EMD boundary by public transportation between 12 pm and 14 pm in minutes	2.51	1.34
Access 15	Log-transformed averaged travel time to private hospitals and clinics within the EMD boundary by public transportation during evening peak (18 pm–20 pm) in minutes	2.63	1.46
Access 16	Log-transformed averaged travel time to public health centers within the EMD boundary by public transportation during morning peak (7 am–9 am) in minutes	2.99	0.54
Access 17	Log-transformed averaged travel time to public health centers within the EMD boundary by public transportation between 12 pm and 14 pm in minutes	3.06	0.60
Access 18	Log-transformed averaged travel time to public health centers within the EMD boundary by public transportation during evening peak (18 pm–20 pm) in minutes	3.22	0.80

**Table 3:** Description of Independent Variables Used in Multilevel Regression Models.

Variables	Description	Mean	S.D.
Old	Log-transformed the density of the population aged over 65 in persons per km^2^ at the SGG (Si/Gun/Gu) level in 2019	5.10	1.80
Bene	Log-transformed the density of people in persons per km^2^ in 2019	3.28	1.91
Immi	Log-transformed the density of immigrants in persons per km^2^ in 2019	2.84	2.14
Female	Log-transformed the density of the female population in persons per km^2^ in 2019	6.12	2.05
Dis	Log-transformed the density of the population with disability classified as the first class in persons per km^2^ in 2018	1.36	1.83
Metro	1 if the administrative boundary is in metropolitan areas (e.g., Seoul, Busan, Daegu, Incheon, Gwangju, Daejon, and Ulsan), 0 otherwise	0.33	0.47
Rural	1 if the administrative boundary is in rural areas (i.e., Eup and Myeon), 0 otherwise	0.40	0.49

**Table 4:** Average Travel Time to Healthcare Services by Car between 5 Clusters.

Cluster	Cluster A	Cluster B	Cluster C	Cluster D	Cluster E	
Degree of Accessibility	Very high	High	Medium	Low	Very Low	**ANOVA**
Observations	637	423	976	1330	88	
Average Travel Time to General Hospital in Minutes						
07 am–09 am	6.65	7.33	8.68	36.36	80.15	***
12 pm–14 pm	7.01	7.59	9.23	37.65	81.66	***
18 pm–20 pm	7.14	7.93	9.36	37.48	92.09	***
Average Travel Time to Private Hospital/Clinic in Minutes						
07 am–09 am	1.80	1.77	2.49	11.13	51.94	***
12 pm–14 pm	1.89	1.81	2.61	11.46	53.90	***
18 pm–20 pm	1.93	1.87	2.65	11.38	71.82	***
Average Travel Time to Public Hospital in Minutes						
07 am–09 am	7.27	8.10	7.83	5.70	10.01	***
12 pm–14 pm	7.71	8.53	8.42	5.82	10.39	***
18 pm–20 pm	7.78	8.88	8.48	5.76	20.46	***

Significance level: * *p* < 0.1; ** *p* < 0.05; *** *p* < 0.01.

**Table 5:** Average Travel Time to Healthcare Services by Public Transportation between 5 Clusters.

Cluster	Cluster A	Cluster B	Cluster C	Cluster D	Cluster E	
Degree of Accessibility	Very high	High	Medium	Low	Very Low	**ANOVA**
Observations	402	1265	432	647	708	
Average Travel Time to General Hospital in Minutes						
07 am–09 am	14.20	15.59	33.37	78.86	105.33	***
12 pm–14 pm	14.60	06.00	35.59	87.13	111.47	***
18 pm–20 pm	14.89	16.26	35.41	95.14	120.00	***
Average Travel Time to Private Hospital/Clinic in Minutes						
07 am–09 am	3.31	4.27	11.76	40.90	70.68	***
12 pm–14 pm	3.32	4.29	12.48	47.20	84.86	***
18 pm–20 pm	3.33	4.30	12.40	51.47	120.00	***
Average Travel Time to Public Health Centers in Minutes						
07 am–09 am	15.59	16.67	21.37	28.56	37.07	***
12 pm–14 pm	16.02	17.16	22.40	32.21	45.03	***
18 pm–20 pm	16.43	17.38	22.65	41.96	83.37	***

Significance level: * *p* < 0.1; ** *p* < 0.05; *** *p* < 0.01.

**Table 6:** Socioeconomic Inequality in Accessibility to Healthcare Services by Car.

Model	OLS 1	OLS 2	OLS 3	OLS 4	OLS 5	OLS 6	OLS 7	OLS 8	OLS 9
DV	Access 1	Access 2	Access 3	Access 4	Access 5	Access 6	Access 7	Access 8	Access 9
	Accessibility to General Hospitals			Accessibility to Private Hospitals and Clinics			Accessibility to Public Health Centers		
	07 am–09 am	12 pm–14 pm	18 pm–20 pm	07 am–09 am	12 pm–14 pm	18 pm–20 pm	07 am–09 am	12 pm–14 pm	18 pm–20 pm
IV	Estimate (Std. Err)	Estimate (Std. Err)	Estimate (Std. Err)	Estimate (Std. Err)	Estimate (Std. Err)	Estimate (Std. Err)	Estimate (Std. Err)	Estimate (Std. Err)	Estimate (Std. Err)
Old	0.372 *** (0.048)	0.372 *** (0.047)	0.386 *** (0.048)	0.171 *** (0.047)	0.166 *** (0.047)	0.192 *** (0.049)	−0.141 *** (0.036)	−0.126 *** (0.035)	−0.094 ** (0.038)
Bene	−0.298 *** (0.037)	−0.283 *** (0.037)	−0.292 *** (0.037)	−0.080 ** (0.036)	−0.080 ** (0.036)	−0.083 ** (0.038)	−0.109 *** (0.028)	−0.114 *** (0.028)	−0.126 *** (0.030)
Immi	0.020 (0.014)	0.019 (0.014)	0.020 (0.014)	−0.010 (0.014)	−0.010 (0.014)	−0.008 (0.015)	−0.00002 (0.011)	−0.0002 (0.011)	0.0003 (0.011)
Female	−0.192 *** (0.050)	−0.185 *** (0.049)	−0.179 *** (0.050)	−0.238 *** (0.049)	−0.238 *** (0.049)	−0.243 *** (0.051)	0.115 *** (0.037)	0.112 *** (0.037)	0.120 *** (0.040)
Dis	−0.001 (0.067)	−0.023 (0.066)	−0.026 (0.068)	0.051 (0.066)	0.053 (0.066)	0.038 (0.069)	0.140 *** (0.050)	0.135 *** (0.050)	0.116 ** (0.054)
Metro	0.040 (0.031)	0.024 (0.031)	0.016 (0.032)	0.007 (0.031)	0.003 (0.031)	−0.008 (0.032)	−0.003 (0.024)	−0.019 (0.023)	−0.030 (0.025)
Rural	1.129 *** (0.029)	1.103 *** (0.029)	1.107 *** (0.030)	1.186 *** (0.029)	1.167 *** (0.029)	1.165 *** (0.030)	−0.207 *** (0.022)	−0.259 *** (0.022)	−0.246 *** (0.024)
Con.	2.311 *** (0.248)	2.311 *** (0.244)	2.252 *** (0.249)	1.678 *** (0.243)	1.745 *** (0.243)	1.695 *** (0.254)	2.126 *** (0.186)	2.160 *** (0.184)	2.024 *** (0.198)
Model Performance									
N	3447	3447	3447	3447	3447	3447	3447	3447	3447
R^2^	0.672	0.672	0.658	0.700	0.697	0.676	0.113	0.146	0.135
Adj. R^2^	0.671	0.671	0.657	0.699	0.697	0.675	0.111	0.145	0.133

Significance level: * *p* < 0.1; ** *p* < 0.05; *** *p* < 0.01. Independent Variable Description: Old: Log-transformed the density of the population aged over 65 in persons per km^2^; Bene: Log-transformed the density of people in persons per km^2^; Immi: Log-transformed the density of immigrants in persons per km^2^; Female: Log-transformed the density of the female population in persons per km^2^; Dis: Log-transformed the density of the population with disability classified as the first class in persons per km^2^; Metro: 1 if the administrative boundary is in metropolitan areas, such as Seoul, 0 otherwise; Rural: 1 if the administrative boundary is in rural areas (i.e., Eup and Myeon), 0 otherwise.

**Table 7:** Socioeconomic Inequality in Accessibility to Healthcare Services by Public Transportation.

Model	OLS 10	OLS 11	OLS 12	OLS 13	OLS 14	OLS 15	OLS 16	OLS 17	OLS 18
DV	Access 10	Access 11	Access 12	Access 13	Access 14	Access 15	Access 16	Access 17	Access 18
	Accessibility to General Hospitals			Accessibility to Private Hospitals and Clinics			Accessibility to Public Health Centers		
	07 am–09 am	12 pm–14 pm	18 pm–20 pm	07 am–09 am	12 pm–14 pm	18 pm–20 pm	07 am–09 am	12 pm–14 pm	18 pm–20 pm
IV	Estimate (Std. Err)	Estimate (Std. Err)	Estimate (Std. Err)	Estimate (Std. Err)	Estimate (Std. Err)	Estimate (Std. Err)	Estimate (Std. Err)	Estimate (Std. Err)	Estimate (Std. Err)
Old	0.141 *** (0.038)	0.141 *** (0.038)	0.129 *** (0.040)	0.092 * (0.049)	0.184 *** (0.050)	0.264 *** (0.053)	−0.124 *** (0.043)	−0.069 (0.046)	0.123 ** (0.059)
Bene	−0.147 *** (0.029)	−0.147 *** (0.030)	−0.161 *** (0.031)	0.032 (0.038)	−0.001 (0.039)	−0.040 (0.041)	−0.071 ** (0.034)	−0.095 *** (0.036)	−0.125 *** (0.046)
Immi	0.023 ** (0.011)	0.031 *** (0.011)	0.047 *** (0.012)	−0.003 (0.015)	0.013 (0.015)	0.034 ** (0.016)	0.005 (0.013)	0.012 (0.014)	0.032 * (0.018)
Female	−0.233 *** (0.039)	−0.249 *** (0.040)	−0.250 *** (0.041)	−0.330 *** (0.051)	−0.444 *** (0.052)	−0.493 *** (0.055)	−0.051 (0.045)	−0.107 ** (0.048)	−0.205 *** (0.062)
Dis	0.093 * (0.053)	0.099 * (0.054)	0.095 * (0.056)	0.040 (0.069)	0.071 (0.071)	0.023 (0.075)	0.168 *** (0.061)	0.178 *** (0.066)	0.057 (0.083)
Metro	−0.053 ** (0.025)	−0.064 ** (0.025)	−0.065 ** (0.026)	−0.071 ** (0.032)	−0.065 * (0.033)	−0.045 (0.035)	−0.010 (0.029)	−0.005 (0.031)	0.018 (0.039)
Rural	1.172 *** (0.023)	1.199 *** (0.024)	1.191 *** (0.025)	1.616 *** (0.030)	1.702 *** (0.031)	1.823 *** (0.033)	0.218 *** (0.027)	0.269 *** (0.029)	0.411 *** (0.037)
Con.	3.995 *** (0.195)	4.108 *** (0.199)	4.238 *** (0.207)	3.208 *** (0.255)	3.497 *** (0.262)	3.590 *** (0.276)	3.846 *** (0.225)	4.002 *** (0.242)	3.919 *** (0.308)
Model Performance									
N	3447	3447	3447	3447	3447	3447	3447	3447	3447
R^2^	0.796	0.799	0.794	0.810	0.822	0.834	0.197	0.235	0.305
Adj. R^2^	0.796	0.799	0.793	0.809	0.822	0.834	0.196	0.234	0.303

Significance level: * *p* < 0.1; ** *p* < 0.05; *** *p* < 0.01. Independent Variable Description: Old: Log-transformed the density of the population aged over 65 in persons per km^2^; Bene: Log-transformed the density of people in persons per km^2^; Immi: Log-transformed the density of immigrants in persons per km^2^; Female: Log-transformed the density of the female population in persons per km^2^; Dis: Log-transformed the density of the population with disability classified as the first class in persons per km^2^; Metro: 1 if the administrative boundary is in metropolitan areas, such as Seoul, 0 otherwise; Rural: 1 if the administrative boundary is in rural areas (i.e., Eup and Myeon), 0 otherwise.

## Data Availability

The two main data sets used in the analysis of this study are publicly available on websites: (1) https://www.ktdb.go.kr/www/selectTrnsportTreeView.do?key=32 (accessed on 5 June 2022) and (2) https://kostat.go.kr/portal/korea/index.action (accessed on 10 June 2022).
